# Benford’s Law Applies to Online Social Networks

**DOI:** 10.1371/journal.pone.0135169

**Published:** 2015-08-26

**Authors:** Jennifer Golbeck

**Affiliations:** University of Maryland, College Park, MD, United States of America; Tianjin University of Technology, CHINA

## Abstract

Benford’s Law states that, in naturally occurring systems, the frequency of numbers’ first digits is not evenly distributed. Numbers beginning with a 1 occur roughly 30% of the time, and are six times more common than numbers beginning with a 9. We show that Benford’s Law applies to social and behavioral features of users in online social networks. Using social data from five major social networks (Facebook, Twitter, Google Plus, Pinterest, and LiveJournal), we show that the distribution of first significant digits of friend and follower counts for users in these systems follow Benford’s Law. The same is true for the number of posts users make. We extend this to egocentric networks, showing that friend counts among the people in an individual’s social network also follows the expected distribution. We discuss how this can be used to detect suspicious or fraudulent activity online and to validate datasets.

## Introduction

Benford’s Law states that, in naturally occurring systems, the frequency of numbers’ first digits is not evenly distributed. Numbers beginning with a “1” are far more common than numbers beginning with “9”—more than six times as frequent. The exact frequency *P* predicted for a digit *d* is given by this formula:

$$\begin{array}{c} P\left(d\right)=\log_{10}\left(1+\frac{1}{d}\right) \end{array}$$

Benford’s Law is frequently used in forensic accounting, where a distribution of first digits that is outside the expected distribution may indicate fraud [1]. Research has also shown that it applies to genome data [2], scientific regression coefficients [3], election data [4, 5], the stock market [6], and even to JPEG compression [7].

We conducted an analysis over five of the most popular social networking websites and found that Benford’s Law applies to the social network structure in all of them. Specifically, the first significant digit (FSD) of users’ friend and follower counts on Facebook, Twitter, Google Plus, Pinterest, and LiveJournal all follow Benford’s Law. Users’ numbers of posts also conform to Benford. To our knowledge, this is the first time Benford’s Law has been applied to social networks. We show that exceptions to this rule can uncover configurations within social media systems that lead to unexpected results.

We also show that, for any individual, the distribution of friend counts within his or her egocentric network also follows Benford’s Law. When the expected distribution is violated, it indicates unusual behavior. A preliminary analysis of over 20,000 Twitter accounts showed that the 100 users whose egocentric networks deviated most strongly from the Benford’s Law distribution were all engaged in suspicious activity.

We discuss how these results lead to the possibility of Benford’s Law being used to detect malicious or irregular behavior on social media. We also show that it could be used to validate the sampling in social media datasets.

## Benford’s Law: Background and Related Work

It was astronomer Simon Newcomb who first formulated what came to be known as Benford’s Law in the 1880s. He noticed that books with logarithm tables showed a lot more wear toward the front, where the numbers beginning with 1 were, than in the back toward the 9s. Concluding that numbers beginning with 1 must be more common, he calculated the probability formula mentioned above.

Physicist Frank Benford noticed the same phenomenon. He validated the observation by collecting naturally occurring numbers from many sources: the surface area of rivers, atomic weights, and numbers appearing in Reader’s Digest [8]. All values followed the pattern. Although they were not a perfect match [9], the principle was established.

The formula for the law, $P(d)={\text{log}}_{10}(1+\frac{1}{d})$, provides a theoretical distribution of expected first digits, shown in Table 1. On the surface, Benford’s Law is quite counterintuitive. Why would numbers beginning with 1 be any more common than those beginning with 9?

**Table 1:** Frequency of first significant digits (FSD) expected by Benford’s Law.

**FSD**	1	2	3	4	5	6	7	8	9
**Frequency**	0.301	0.176	0.125	0.097	0.07918	0.067	0.05799	0.051	0.046

Nevertheless, the law holds across many variations in measurement [10]. Temperatures that follow Benford’s Law do so regardless of whether they are measured in Fahrenheit, Celsius, or Kelvin. Distances follow whether measured in miles, kilometers, or smoots. Most persuasively, Hill provided a proof for the Benford’s Law in 1995 [11]. As a simple demonstration for skeptics, he suggests that they jot down all the numbers that appear on the front pages of several newspapers, or randomly select data from the Farmer’s Almanac [6].

Benford’s Law describes all these naturally occurring sets of numbers, and more. Specifically, some applications of Benford’s Law are more relevant to our work. Benford often applies to systems that follow a power law distribution [12]. Power laws are commonly found in social network structures [13] and social media [14]. Although no one has yet investigated how well Benford’s Law describes social networks (online or offline) or social media, it has been shown to describe online human behavior through price distributions in eBay auctions [15].

## Data, Data Sets, and Collection

We analyzed data from five major social networking websites: Facebook, Twitter, Google Plus, Pinterest, and LiveJournal.

We collected the number of friends in each network and followers when appropriate. On Google Plus, Twitter, and LiveJournal, we also had access to egocentric network data. For each user, we obtained a list of friends and the count of outgoing edges for each of those friends. With this data, we could analyze the distribution of FSDs for an individual person’s social network

On Twitter and Pinterest, we also had access to the number of posts each person made. This provides another interesting insight into the general patterns of behavior on social media and whether Benford’s Law applies. We collected some of these datasets ourselves and used other datasets that had been created by others. The following sections detail our process with each network.

### Facebook

We accessed user profiles using the Facebook Graph API with requests for friend counts of a numeric Facebook user ID. Once we accessed a user’s data, we incremented the user ID by 10,000 and make the next request. If there was no data available for a given user, we incremented the user ID by 1 and tried the next person until we found a match. We collected friend counts for 18,298 users.

### Twitter

We collected the numbers of followers and “friends” (i.e. people the user is following) the user had, and the number of people each of those friends were following. This allowed us to analyze the distribution of FSDs within egocentric social networks. In addition to this network data, we collected the number of status updates for each user.

Although there are existing Twitter social network datasets online, we collected our own data in this project in order to work with non-anonymized users so we could later analyze their account activity.

We accessed data via the Twitter API, using users’ numeric Twitter user ID. Our process was to access a user’s data and then increase the user ID by 50,000. This gave us a fairly uniform distribution of users, and we were able to collect the data in a reasonable amount of time (over a few weeks) given Twitter’s API limits.

If an a user ID was protected or not linked to an account, we incremented user the ID by 1 and tried the next person until we found a match.

Because we considered counts for friends, followers, and status updates, we only included users in our sample who had at least one of each. This allowed us to consider that same set of users for all three attributes.

For egocentric network analysis, we only included users who had at least 100 friends so the distribution of FSDs would be measured over a reasonably large sample.

Our final dataset had 78,225 users. For 21,135 of these, we also had egocentric networks with friend, follower, and status counts for all the people they were following.

### Google Plus

The Google Plus network [16] is part of the Stanford Network Analysis Project (SNAP) datasets, The social network is provided as an adjacency list. We made one pass through the “combined” dataset, counting the number of friends a person had. The network is directed, so we counted outgoing edges. In addition to using the friend counts for each person, we were able to get the friend count for each of their friends, thus allowing us to construct a FSD distribution for each egocentric social network. After processing, we had data for 72,271 users.

### Pinterest

The Pinterest data was provided as part of the Social Curation Dataset [17]. We used the “Pinterest User Information” data, which contained follower, following, and pin (i.e. post) counts. After filtering out users with no followers or pins, we had data for 39,586,033 users.

### LiveJournal

LiveJournal Dataset [18, 19] is one of the SNAP datasets. We followed the same processing procedure as we used for the Google Plus dataset. After processing, we had data for 4,307,491 users.

## Results

**Fig 1:**
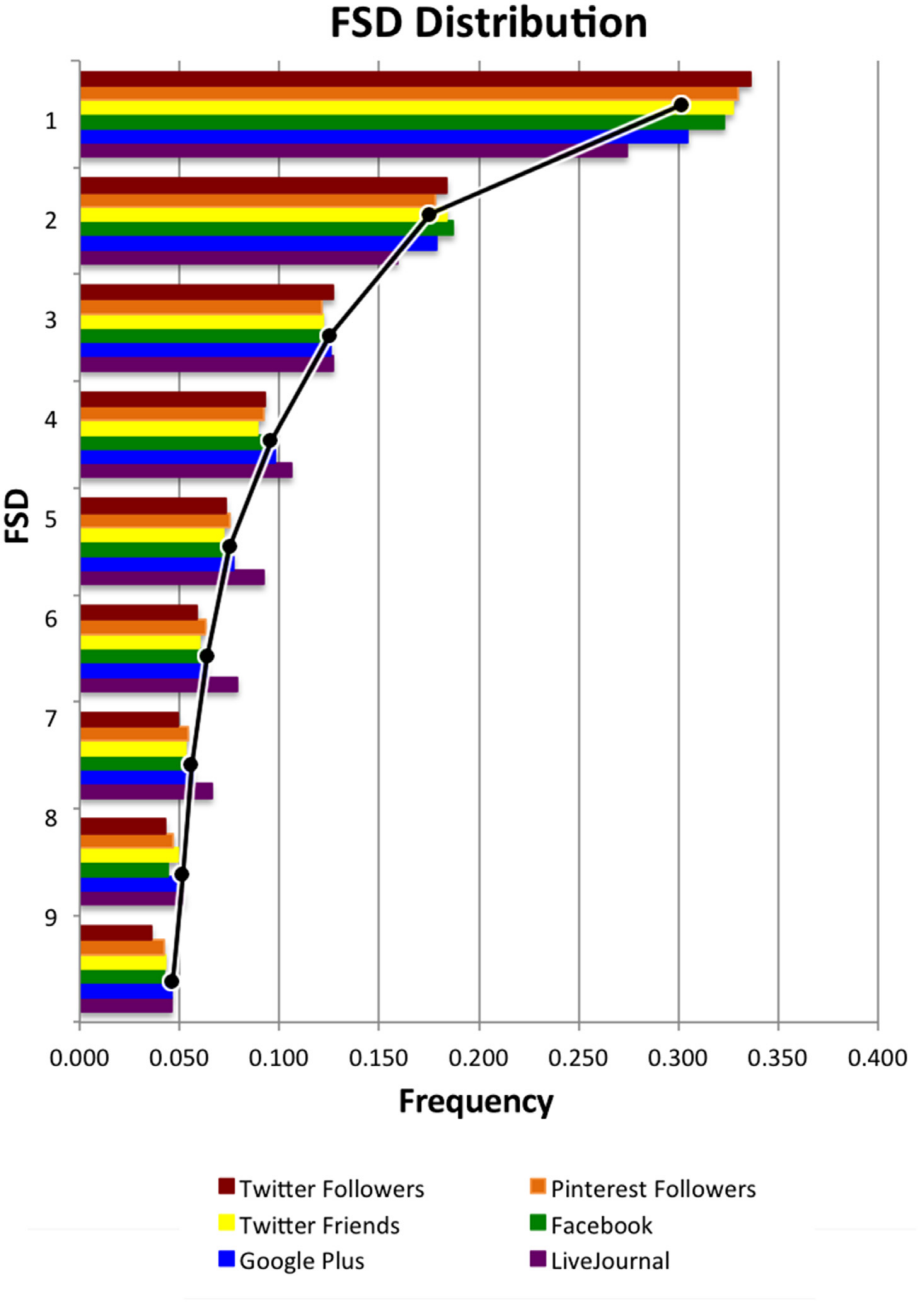
Distribution of first significant digits for Twitter (friends and followers), Google Plus, Pinterest followers, Facebook, and LiveJournal. The black trend-line shows the value predicted by Benford’s Law for each FSD.

We found that the distribution of FSD among friends in all five datasets closely followed the values expected from Benford’s Law, with one interesting exception: the Pinterest following relationship. We discuss this later, but we will set this aside for now. Fig 1 shows the distribution of FSDs for each of the six datasets.

For Facebook, Google Plus, and LiveJournal friends, Twitter friends and followers, and Pinterest followers, all the distributions of FSDs followed Benford’s Law. Note that with datasets of this size, it is not appropriate to conduct a statistical hypothesis test for goodness of fit; over tens of thousands or millions of people, even a very tiny deviation would cause us to reject the null hypothesis. Furthermore, conformance with Benford’s Law has never been about a perfect statistical match to the predicted values—not even in Benford’s original work on the subject [8, 9]. Rather, the relative frequencies of FSDs are the guiding principle.

Pearson correlations are a common way to measure how closely a distribution adheres to Benford’s Law [20–22]. The correlation between the FSDs of the friend–follower counts and what Benford’s Law predicts are extremely strong. As shown in Table 2, all the *r* values are > 0.990. We also ran Kolmogorov–Smirnov tests to check the fit of the data with the Benford’s Law distribution. The p-values, which indicate the probability that the social network’s FSD distribution is the same as Benford’s, are also shown in Table 2. These values are all > 0.97.

**Table 2:** Pearson Correlation and p-values from Kolmogorov–Smirnov Tests between the distribution of first significant digits of friend/follower distributions of various social networks and values predicted by Benford’s Law.

**Site**	**Total Users**	**Correlation**	**KS p value**
**Google Plus**	72,271	0.9999	0.9794
**Facebook**	18,298	0.9996	0.9794
**Twitter Friends**	78,225	0.9990	1.000
**Twitter Followers**	78,225	0.9998	0.9895
**Pinterest Followers**	39,586,033	0.9989	1.000
**LiveJournal**	4,307,491	0.9695	0.9895

Other user behavior also fit Benford’s Law as well. We had data for the number of posts users made on Pinterest (number of pins) and Twitter (number of tweets). In both cases, correlation with Benford’s predictions was extremely high: 0.9998 and 0.9960, respectively.

**Fig 2:**
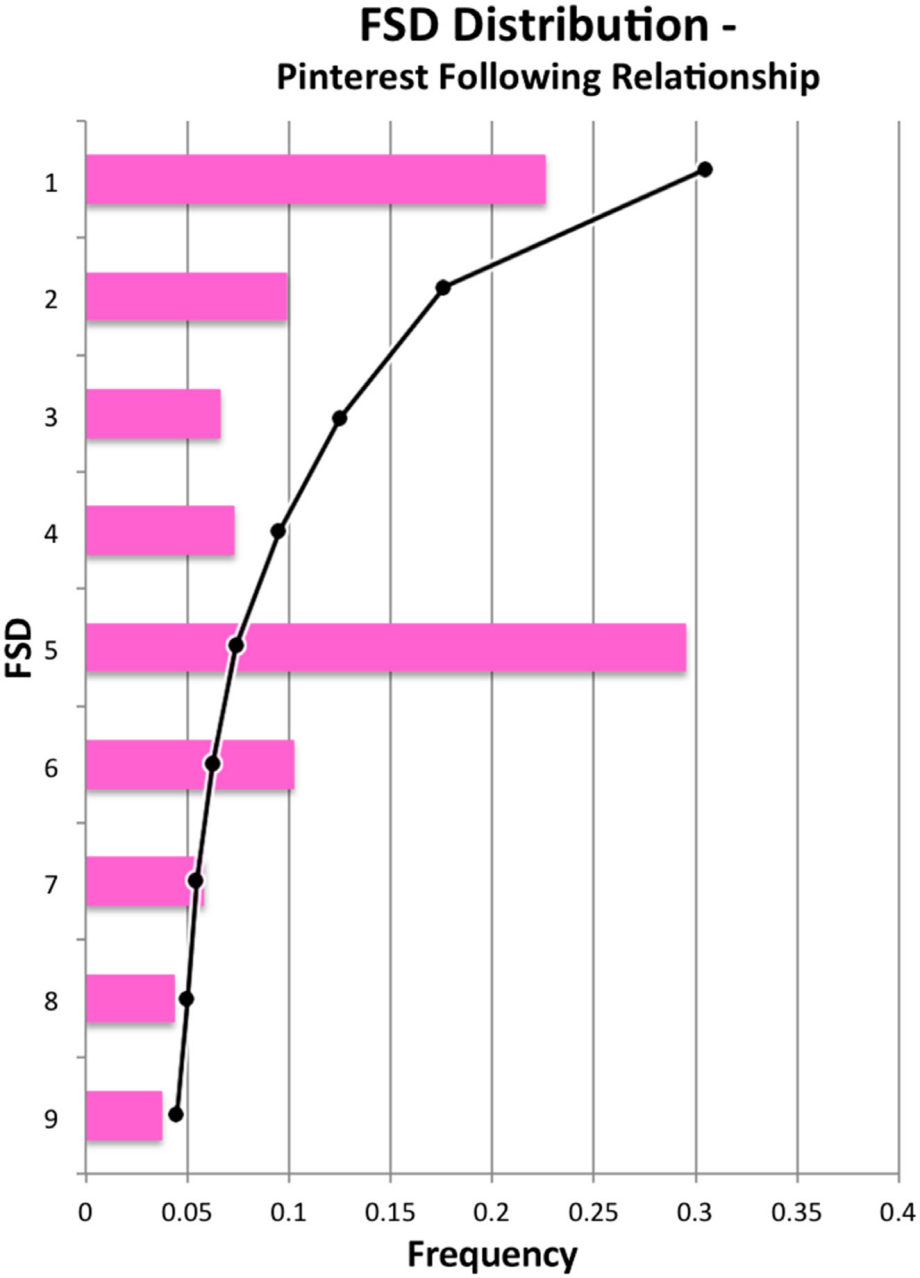
Distribution of first significant digits for Pinterest users’ following relationships. The black trend-line shows the value predicted by Benford’s Law for each FSD.

However, we mentioned above that one dataset did not follow Benford’s predictions. On Pinterest, users have both a follower count, which represents incoming social connections, and a following count for outgoing edges. The follower count is what we presented above, and it follows the expected distribution. The *following* count did not adhere to Benford’s Law (see Fig 2). The percentages are very far off the law’s prediction, and the dominance of FSDs of 5 is especially striking.

Is this simply an exception to the rule, or is something else going on? When Benford’s Law is applied in forensic accounting, auditors know to look for explanations of data that appears unusual. For example, a company may have a high percentage of FSDs of 3, not because anything fraudulent is happening, but because they happen to frequently purchase an item that costs $39.99.

We investigated this issue on Pinterest more deeply and found the explanation for the frequent 5s. When new users sign up for Pinterest, they are prompted to choose “interests” to follow. Users *must* select at least five before continuing with the registration process. This creates at least five initial following relationships for users. Though users can go in and later delete those follows, few do, and this initiation process affects the entire distribution of FSDs. When we looked at the edges in the opposite direction (considering incoming follower edges rather than outgoing), the FSDs adhered to Benford’s Law, as shown above.

This exposes an important point about applying Benford’s Law: it can be violated when there is external influence over people’s natural behavior. In the Pinterest case, we discovered the influence was an artifact of the system configuration.

Benford’s Law extends to second digits, as well. The distribution is much flatter, ranging from 0.1197 for the frequency of 1s to 0.0850 for the frequency of 9s. Our networks agreed with the second digit distribution quite well. The Pearson correlations for all five networks, including Twitter friend counts and follower counts, are all > 0.97. The Kolmogorov–Smirnov p-values are > 0.98 for Twitter friends, Facebook, Pinterest, and Google Plus.

This second digit analysis eliminated the frequent 5s artifact seen in Pinterest. The Kolmogorov–Smirnov p-values for LiveJournal and Twitter follower counts are a bit less impressive (0.79 and 0.76, respectively), though some existing literature suggests this might be the expected with the flatter distribution of the second digit [3, 23].

### Extension to Egocentric Networks

The adherence to Benford’s Law carries through into FSD distributions within individual egocentric networks. Using data from Twitter, Google Plus, and LiveJournal, we selected individuals with at least 100 friends, and then obtained the number of social connections that each of those friends had. We then determined FSD distributions in the friend-of-friend counts of each egocentric network. Overall, the vast majority of egocentric networks conformed to Benford’s Law.

On Google Plus, 91.5% of users’ egocentric networks’ FSD distributions had a correlation of over 0.9 with Benford’s Law predictions. This was true for 85.1% of LiveJournal egocentric networks.

In the Twitter data, 89.7% of users had a correlation of over 0.9. Of our 21,135 users, only 170 (< 1%) had a correlation under 0.5.

Since we had non-anonymized data for Twitter, we were able to investigate these accounts with low correlations. Nearly every last one of the 170 accounts mentioned above appeared to be engaged in suspicious activity. Some accounts were spam, but most were part of a network of Russian bots that posted random snippets of literary works or quotations, often pulled arbitrarily from the middle of a sentence. All the Russian accounts behaved the same way: following other accounts of their type, posting exactly one stock photo image, and using a different stock photo image as the profile picture. While we are currently investigating the purpose of these bot accounts’ existence, their deviation from Benford’s Law made it quite easy to identify their highly unusual behavior. Of the 170 accounts, only 2 seemed to belong to legitimate users.

**Fig 3:**
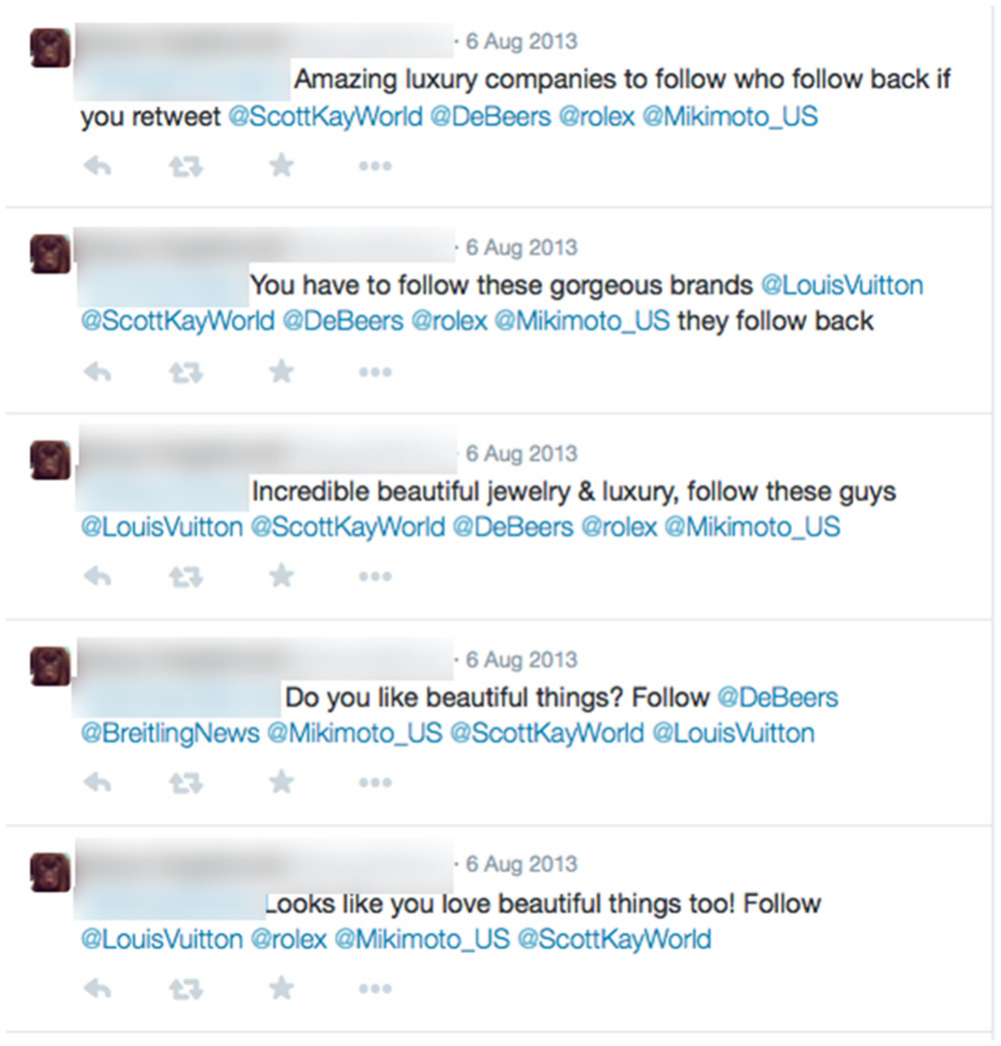
Example posts from one of the spam accounts we detected.

**Fig 4:**
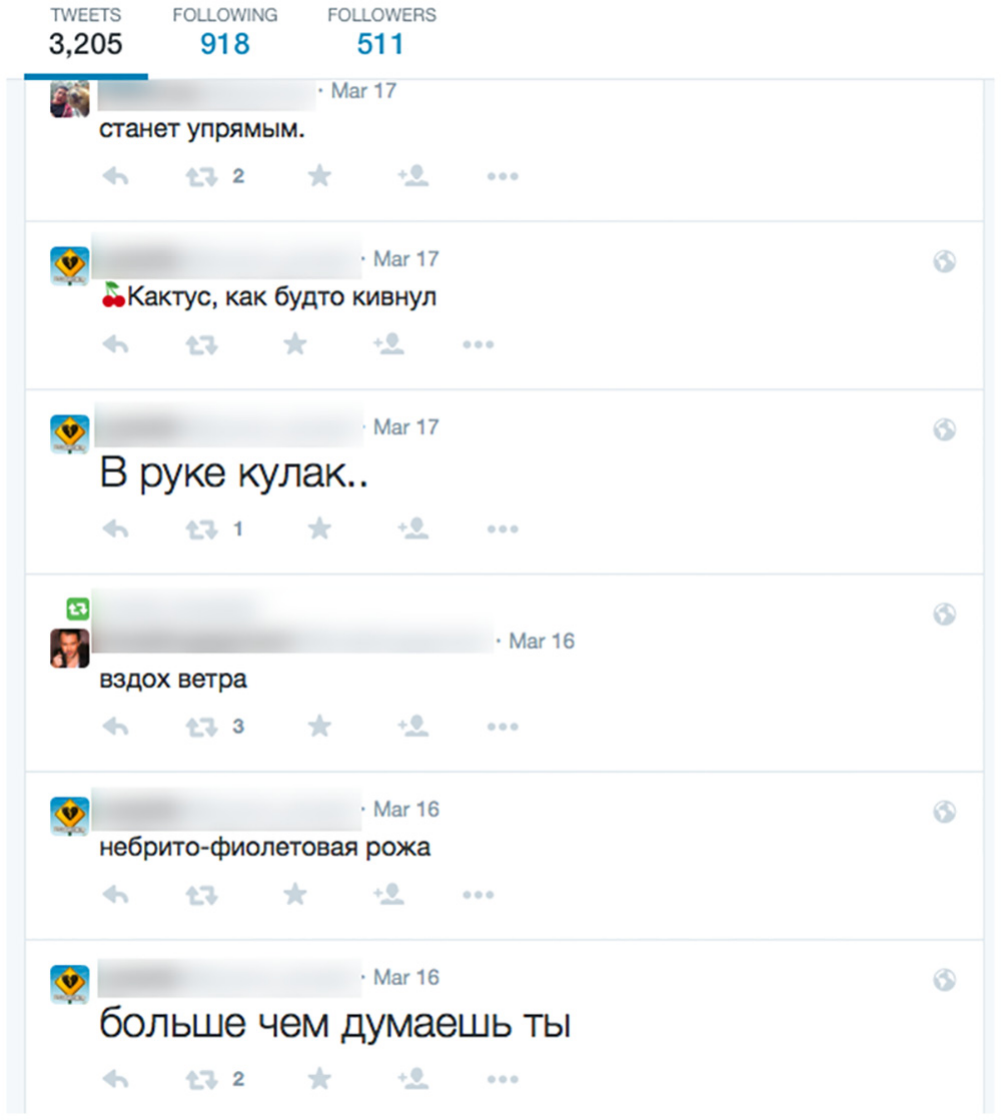
Example posts from one of the Russian bot accounts we detected.

Figs 3 and 4 show examples of a spam account and Russian bot account detected by this method.

## Discussion and Conclusions

We have shown that Benford’s Law applies to relationships in online social networks. This is true for social networking sites as a whole, and for individual users’ egocentric networks. Data from Twitter and Pinterest also suggest that it applies to the number of posts users make on social media sites, as well. In the one network where Benford’s Law did not hold, closer inspection revealed that it due to a feature of the system that altered users’ behavior.

Next are some applications for these results, followed by some closing thoughts on the work.

### Applications

First, Benford’s Law can be used to detect users who are behaving in unexpected ways. As we found in our Twitter dataset, the vast majority of accounts that strongly deviated from the expected FSD distributions were engaged in unusual behavior. As is the case with forensic accounting investigations using Benford’s Law, a deviation does not necessarily mean there is fraud happening. Given the large number of users on social media, it would be statistically unusual to have *no* accounts that naturally deviate from expected patterns; rather, deviation from a Benford distribution can flag accounts for additional review.

These insights can also be used to validate experimental datasets. It is often the case that data can be hard to collect from social media sites, especially when researchers are looking for detailed personal information. Truly random or representative sampling is difficult to do—and essentially impossible when connected components of a social network are important to the analysis. This raises the question as to whether the sample of accounts collected by a research team seriously deviates from normal patterns. While Benford’s Law only addresses one aspect of expected behavior, major differences between a sample’s FSD distribution and Benford’s Law could indicate serious sampling problems.

We tested this by analyzing the FSD distributions on a number of datasets collected for various projects and experiments.

We randomly selected 50 Twitter-based networks from the NodeXL Graph Gallery (http://nodexlgraphgallery.org). These were all generated by collecting the networks of users who had tweeted a given search term. For each graph, we analyzed friend, follower, and tweet counts for the users in each dataset. On all graphs and each of the three measures, the Pearson correlation with Benford’s expected values was > 0.990. This shows that, structurally, the networks look like we would expect.

We found similarly strong correlations and agreement with Twitter data collected for a research project that posted a survey on a popular psychology website. Subjects included their Twitter IDs in their survey responses. The distributions of FSDs for friends, followers, and tweets all correlated with Benford’s Law distributions with *r* > 0.990 and very close values.

However, not all datasets were a good match. One Facebook dataset of 151 users had a Pearson correlation of 0.761, and there were large differences between the predicted and actual frequencies. All but two FSDs saw deviations over 25% from expected values, and some saw deviations over 80%. This was true on another Facebook dataset with 220 users (supplied by a colleague). In this example, friend counts were self-reported, and 94.5% of those began with a 1—more than triple the expected 30.1%.

Such deviations do not necessarily imply a problem with the data; indeed, this distribution may be irrelevant to the analysis being performed. However, it hints that the subjects are not reflecting an expected distribution, and thus may vary from the larger population in other ways. Further research is needed to understand the implications of deviation in experimental samples.

There is a growing understanding of the subtle patterns of natural behavior which humans have difficulty replicating in unnatural circumstances. The applicability of Benford’s Law to social media is a new tool for analyzing user behavior, understanding when and why natural deviations may occur, and ultimately, detecting when abnormal forces are at work.
